# Phosphorylation mediated regulation of RNA splicing in plants

**DOI:** 10.3389/fpls.2023.1249057

**Published:** 2023-09-14

**Authors:** Maria Camila Rodriguez Gallo, R. Glen Uhrig

**Affiliations:** ^1^ University of Alberta, Department of Biological Sciences, Edmonton, AB, Canada; ^2^ University of Alberta, Department of Biochemistry, Edmonton, AB, Canada

**Keywords:** phosphorylation, protein kinases, RNA splicing, proteomics, regulation

## Abstract

For the past two decades, the study of alternative splicing (AS) and its involvement in plant development and stress response has grown in popularity. Only recently however, has the focus shifted to the study of how AS regulation (or lack-thereof) affects downstream mRNA and protein landscapes and how these AS regulatory events impact plant development and stress tolerance. In humans, protein phosphorylation represents one of the predominant mechanisms by which AS is regulated and thus the protein kinases governing these phosphorylation events are of interest for further study. Large-scale phosphoproteomic studies in plants have consistently found that RNA splicing-related proteins are extensively phosphorylated, however, the signaling pathways involved in AS regulation have not been resolved. In this mini-review, we summarize our current knowledge of the three major splicing-related protein kinase families in plants that are suggested to mediate AS phospho-regulation and draw comparisons to their metazoan orthologs. We also summarize and contextualize the phosphorylation events identified as occurring on splicing-related protein families to illustrate the high degree to which splicing-related proteins are modified, placing a new focus on elucidating the impacts of AS at the protein and PTM-level.

## 1 Introduction

Alternative splicing (AS) is of particular importance for plants, with upwards of ~40-80% of multi-exonic genes undergoing AS (Filichkin et al., 2010; Marquez et al., 2012; Thatcher et al., 2014; Chen et al., 2020; Liu et al., 2022). Correspondingly, plants possess a wide range of spliceosome-related proteins, of which, serine/arginine-rich (SR) proteins and heterogeneous nuclear ribonuclear proteins (hnRNPs) function as positive and negative regulators of RNA splicing, respectively (Barta et al., 2010; Busch and Hertel, 2012; Erkelenz et al., 2013). Many of the genes encoding plant SR proteins are themselves alternatively spliced in response to wide-range of environmental changes, including: changes in light (Filichkin et al., 2010; Petrillo et al., 2014; Tognacca et al., 2019), temperature (Calixto et al., 2018; Li et al., 2021c; Ling et al., 2021), osmolarity (Tanabe et al., 2007; Ding et al., 2014; Albaqami et al., 2019), amongst others (Lazar and Goodman, 2000; Isshiki et al., 2006; Hartmann et al., 2018), with these AS events found to confer stress tolerance in an isoform-dependent manner (Albaqami et al., 2019). Examination of SR protein over-expression and loss-of-function plant lines have shown a variety of developmental phenotypes (Ishizawa et al., 2019; Xu et al., 2019; Lee et al., 2020b) and impacts on gene expression (Hartmann et al., 2016; Wu et al., 2016; Yan et al., 2017), with many of these studies uncovering developmental ramifications as a result of dysregulated AS. However, the ways in which AS is regulated through post-translational modifications (PTMs), such as phosphorylation, has only recently become of interest.

In human cells, AS regulates essential functions such as autophagy (Paronetto et al., 2016; Lv et al., 2021), apoptosis (Singh et al., 2016; Kędzierska and Piekiełko-Witkowska, 2017; Stevens and Oltean, 2019), protein localization (Link et al., 2016), and transcription factor activity (Chen et al., 2022), amongst others (Baralle and Giudice, 2017). Therefore, it is no surprise that AS dysregulation results in several medical conditions including: cancer (Da Silva et al., 2015; Wang et al., 2016), heart disease (Liu et al., 2019; Hasimbegovic et al., 2021), neurological disorders (Low et al., 2021; Zhang et al., 2022; Nishanth and Jha, 2023) and multiple genetic disorders (Maule et al., 2019; Ajiro et al., 2021; Jiang and Chen, 2021). Hence in humans, PTM regulation and the signaling pathways governing AS, have been extensively studied, offering opportunities for comparative analysis of new findings being made in plants.

Comparative analyses of human and plant AS regulation have highlighted the largely conserved functionality of AS across eukaryotes, while also revealing unique AS regulation specific to plants (Chaudhary et al., 2019). In both humans and plants, phosphorylation of SR proteins has been found to induce nucleocytoplasmic shuttling (Rausin et al., 2010; Botti et al., 2017; Park et al., 2017), to initiate binding on pre-mRNA (Zhou and Fu, 2013), and facilitate spliceosome assembly (Saha and Ghosh, 2022). In humans, the interactive networks between splicing-related protein kinases and their SR protein substrates are an active area of research, revealing roles in the regulation of vascular endothelial growth factor A (VEGF-A) signaling (Li et al., 2021b), protein kinase B (AKT)/ERK pathways (Zhou et al., 2012), along with the targeting of rapamycin complex 1 (mTORC1)/ribosomal S6 kinase 1 (S6K1) (Lee et al., 2018) pathway; all of which involve human SRPK (HsSRPK) phosphorylation of SR proteins. CDC2-LIKE KINASEs (CLKs), alongside HsSRPK1, have also been shown to be involved in SR protein mediated AS (Aubol et al., 2003; Ngo et al., 2005; Kulkarni et al., 2017). However, in plants, the intricate links between signal transduction, protein phosphorylation, and AS is just beginning to emerge.

In this mini-review, we describe the current state of splicing-related protein kinase research in plants, relating this knowledge to our established understanding of these proteins kinases in humans. We then examine the extent to which splicing-related proteins are phosphorylated and touch upon AS dysregulation in plants. Finally, we briefly discuss what is next for understanding plant AS from a protein-centric perspective and the implications behind PTM-level regulation.

### 1.1 Splicing-related protein kinases: An overview

Splicing-related protein kinases are conventionally categorized by their ability to phosphorylate splicing factors or components of the spliceosome. Here we summarize the roles and current understanding of the three major splicing-related protein kinase families studied in plants, focusing on the model plant *Arabidopsis* where most of the recent research has emerged.

#### 1.1.1 Serine arginine protein kinases

The *Arabidopsis* SRPK family (AtSRPKs) consists of five members divided into two groups: Group I (SRPK1: AT4G35500, and SRPK2: AT2G17530) and Group II (SRPK3: AT5G22840, SRPK4: AT3G53030, SRPK5: AT3G44850) SRPKs (Rodriguez Gallo et al., 2022). These AtSRPK groupings first become clear with the emergence of spermatophytes, suggesting duplication of the family early in the land plant lineage. SRPK peptide sequences are characterized by a bi-partite kinase domain separated by a spacer region, which is conserved across both the animal and plant kingdoms. The SRPK spacer domain has been found to be required for the nucleocytoplasmic shuttling of HsSRPKs, but not necessary for their kinase activity (Ding et al., 2006; Koutroumani et al., 2017; Sigala et al., 2021). Nonetheless, the presence of the spacer domain has been shown to increase HsSRPK phosphorylation rate by facilitating nucleotide release (Plocinik et al., 2011; Aubol et al., 2012). Although the function of the spacer domain of AtSRPKs remains to be determined, it most likely aids in nucleocytoplasmic shuttling similar to its human orthologs as localization experiments of Group II AtSRPKs have demonstrated both nuclear and cytoplasmic localizations (Wang et al., 2023).

HsSRPK have been implicated in various developmental and stress-related pathways. Similarly, AtSRPKs seem to be involved in a variety of biological processes. For example, AtSRPK1 seems to be stress-induced due to its transcriptional up-regulation under various abiotic stresses (cold, heat, osmotic, salt) (Rodriguez Gallo et al., 2022). Further, all AtSRPKs exhibit diel regulation, with peak transcriptional expression occurring mid-night (ZT18) in seedlings, suggesting that AtSRPKs may be a part of circadian regulated processes or involved in circadian mediated AS events. Accordingly, Group II AtSRPK loss-of-function lines displayed a late-flowering phenotype and an up-regulation of FLOWERING LOCUS C (FLC) gene expression; the major negative regulator of flowering (Wang et al., 2023). In the same study, Group II AtSRPKs were implicated in the phosphorylation of a number of SR proteins and beyond, including proteins involved in ribosome biogenesis, abiotic stress, hormone signaling and carbohydrate responses. The authors found phosphorylation motifs ‘xxxxxxSPxxxxx’ and xxxxSxSxxxxxx’ to be enriched amongst differentially abundant phosphorylation events in Group II deficient (*sprk3 4 5*/*sprkii-1*) plants and suggested they may be Group II specific phosphorylation motifs.

#### 1.1.2 Arabidopsis Fus3 complement

There are three members comprising the AFC family in *Arabidopsis*: AFC1 (AT3G53570), AFC2 (AT4G24740), and AFC3 (AT4G32660). AFCs belong to the family of LAMMER kinases, which are characterized by a conserved ‘AHLAMMERILG’ motif in their catalytic kinase domain that is important for substrate recognition (Lee et al., 1996; Kang et al., 2010) as well as their dual tyrosine and serine/threonine kinase activity profile (Ben-David et al., 1991; Yun et al., 1994). In humans, the CLKs represent the AFC orthologs of plants and have been shown to phosphorylate a multitude of substrates, including SR proteins (Ngo et al., 2005; Varjosalo et al., 2013). CLKs bind to SR proteins but lack the mechanism to release phosphorylated SR proteins, requiring an HsCLK/HsSRPK complex for the release of SR proteins (Aubol et al., 2016; Aubol et al., 2018). In *Arabidopsis*, AFCs have been found to phosphorylate plant SR proteins *in vitro* (Lin et al., 2022), however, the extent to which AFCs phosphorylate non-SR proteins remains unknown.

Phylogenetic analysis of the photosynthetic eukaryote AFCs indicates that the AFC3 group diverged in gymnosperms, while the AFC1 and AFC2 groups emerged later with the evolution of monocots, suggesting that these AFCs may perform non-redundant functions specific to flowering plants (Rodriguez Gallo et al., 2022). To date, AtAFCs have been implicated in thermoregulation, of which AtAFC2 controls high-temperature AS, with *afc2* loss-of-function plants exhibiting aberrant splicing patterns under high temperatures (Lin et al., 2022). Furthermore, AtAFC2 gene expression in shoot tissue is significantly up-regulated under cold stress (Rodriguez Gallo et al., 2022). Connections have also been drawn between temperature, flowering, and AS, with the major spliceform of FLOWERING LOCUS M (FLM) contributing to temperature-responsive flowering in *Arabidopsis* (Capovilla et al., 2015; Jin et al., 2022). Furthermore, *Arabidopsis* splicing factor 1 (SF1) interacts with FLM pre-mRNA in a temperature-dependent manner, inducing the production of FLM-β transcripts, and thus modulating flowering time in response to temperature fluctuations (Lee et al., 2020b). Similarly, the metazoan CLKs also have roles in temperature-dependent AS, whereby lower body temperatures activate HsCLKs, resulting in high SR protein phosphorylation both *in vitro* and *in vivo* (Haltenhof et al., 2020). The same study also connects CLK temperature-dependent activity with the circadian-regulation of internal body temperature. Similarly, AtAFCs are also expressed in a diel manner, with peak expression occurring mid-night (ZT18) (Rodriguez Gallo et al., 2022).

#### 1.1.3 Pre-mRNA processing factor 4 protein kinases

The last major family of characterized splicing kinases are the PRP4Ks. There are three members to the *Arabidopsis* PRP4K family: PRP4Ka (AT3G25840), PRP4kb (AT1G13350), and PRP4Kc (AT3G53640). PRP4Ks were the first protein kinases to be characterized to have a regulatory impact on mRNA splicing in both fungi and mammals (Ltzelberger and Käufer, 2012). HsPRP4K is encoded by a single gene (*PRPF4B)* and is a snRNP-associated kinase. Similar to HsCLKs, HsPRP4K is also a dual-specificity kinase, but unlike the other two families of splicing-related protein kinases, HsPRP4K has been found to associate with major spliceosome proteins (Dellaire et al., 2002) and is required for the formation of the early spliceosome (Schneider et al., 2010). In humans, HsPRP4K plays an essential role in ovarian and other epithelial cancers, with a reduction in HsPRP4K levels leading to anoikis sensitivity (Corkery et al., 2018). To date, our understanding of PRP4Ks across plants is lacking, with only *atprp4ka* loss-of-function plants being phenotypically and biochemically characterized. Here, phosphoproteomic data identified multiple SR splicing factors (e.g. AtSR30, AtRS41, AtRS40, AtSCL33, and AtSCL30A) as possessing significant changes in their phosphorylation status compared to wild-type plants (Kanno et al., 2018).

### 1.2 Phosphorylation of splicing-related proteins

#### 1.2.1 Phosphorylation abundance

**Figure 1:**
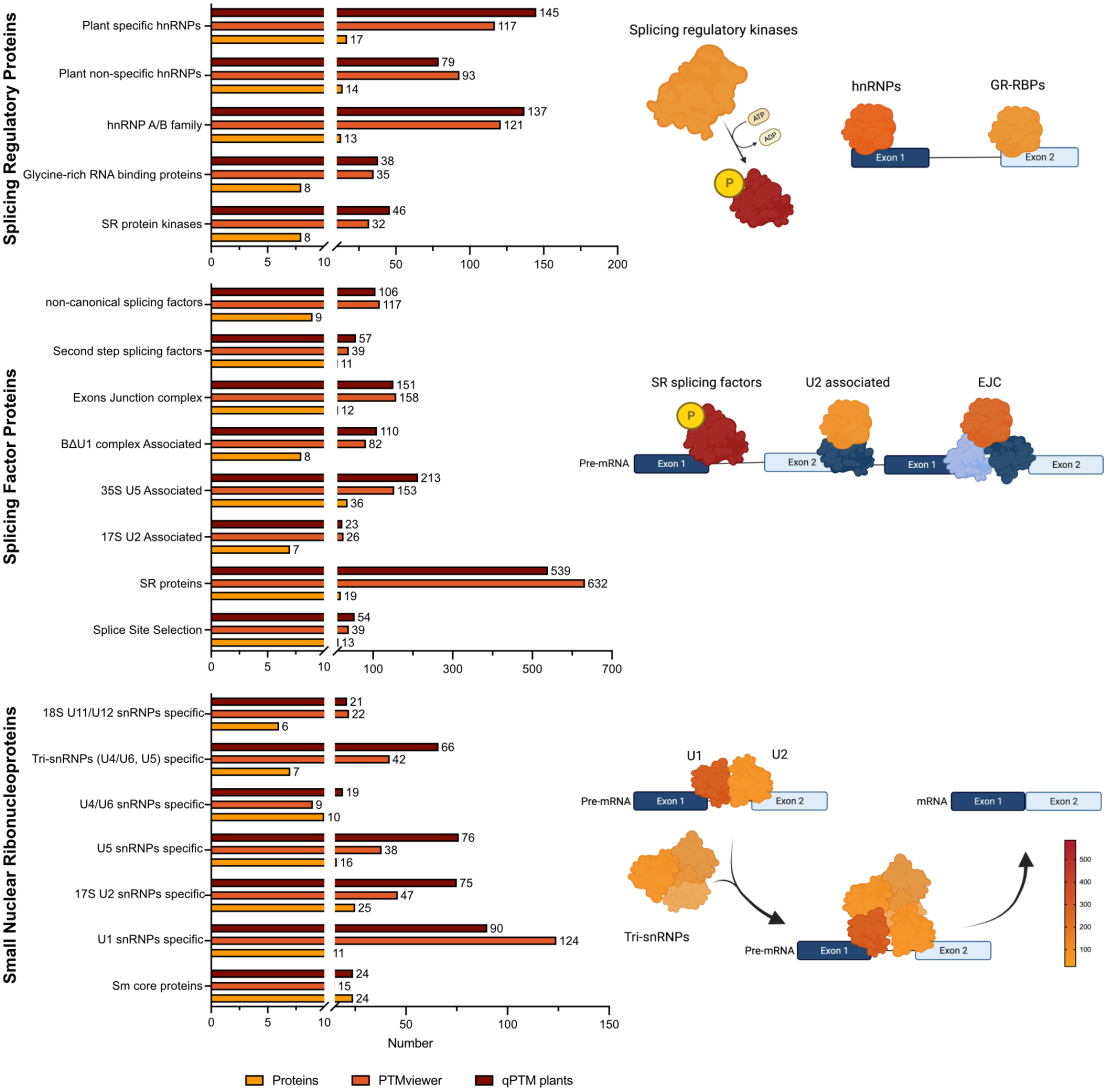
Number of unique protein phosphorylation events identified on splicing-related proteins in *Arabidoposis.* Identified phosphosites were collected from PTMviewer (Willems et al., 2019; https://www.psb.ugent.be/webtools/ptm-viewer/index.php) and qPTM (Xue et al., 2022; http://qptmplants.omicsbio.info/). Selection and categorization of splicing-related proteins were acquired from plantSPEAD (Chen et al., 2021; http://chemyang.ccnu.edu.cn/ccb/database/PlantSPEAD/index.php) and the number of proteins related to each family is plotted. Number of phosphorylation events for select protein families were converted to a colour intensity gradient.

The phosphorylation state of SR proteins can change their activity (Xiang et al., 2013; Keshwani et al., 2015), localization (Stankovic et al., 2016), interaction with other proteins and/or RNA to initiate RNA splicing reactions (Kim et al., 2015). Further, *Arabidopsis* splicing-related proteins have been reported to be extensively phosphorylated in large-scale phosphorproteomic studies (De La Fuente Van Bentem et al., 2006; Marondedze et al., 2016; Mehta et al., 2021). Using plant SPEAD (Chen et al., 2021; http://chemyang.ccnu.edu.cn/ccb/database/PlantSPEAD/index.php), in conjunction with PTM containing databases: PTMviewer (Willems et al., 2019; https://www.psb.ugent.be/webtools/ptm-viewer/index.php) and qPTM plants (Xue et al., 2022; http://qptmplants.omicsbio.info/), the extent to which diverse splicing-related protein families are phosphorylated highlights the need to resolve the function of these regulatory events (**Figure 1**).

In *Arabidopsis*, studies show that the most highly phosphorylated splicing-related proteins are plant specific hnRNPs and the A/B hnRNP family, followed by plant non-specific hnRNPs (**Figure 1**). The hnRNPs were originally discovered by electron micrographs (Gall, 1956) in metazoans and in the years following, were characterized biochemically (Samarina et al., 1966), and then categorized for their binding to nascent transcripts (Beyer et al., 1977). The hnRNPs are involved in a diverse set of processes such as telomere maintenance (Kwon and Chung, 2004; Lee and Kim, 2010; Shishkin et al., 2019), transcription (Li and Liu, 2010; Rauch et al., 2010; Molitor et al., 2016), and pre-mRNA splicing (Tange et al., 2001; Dreyfuss et al., 2002; Streitner et al., 2012; Geuens et al., 2016). Moreover, human hnRNPs undergo nucleocytoplasmic shuttling which has been proposed to be a way of transporting mRNA to the cytoplasm (Beyer et al., 1977; Yeap et al., 2019; Dabral et al., 2020). In the context of RNA splicing, hnRNPs are antagonistic partners to SR splicing factors, where upon binding to splicing silencing sequences on the pre-mRNA, function to repress the formation of early spliceosome (Wang et al., 2004; Matlin et al., 2005; Rahman et al., 2015; Lin et al., 2020). Due to their involvement in the multiple stages of mRNA transcription, maturation, and shuttling, their regulation must be finely tuned and as such, a high-degree of phosphorylation could be expected.

Interestingly, SR proteins have almost five times more phosphorylation events than any other splicing factor protein group in *Arabidopsis* (**Figure 1**). In humans, SR proteins play crucial roles in multiple stages of mRNA maturation, including: splice site selection (Jia et al., 2019; Li et al., 2021a), recruitment of spliceosome proteins (Cho et al., 2011), facilitating mRNA transport to the cytosol (Müller-McNicoll et al., 2016; Jeong, 2017), and mRNA stability (Howard and Sanford, 2015; Grosse et al., 2021). They serve as key determinants of specificity and are believed to integrate multiple signaling pathways mediated by phosphorylation through SRPKs. Human SR proteins are categorized as containing one or two RNA-recognition motifs (RRMs) at their N-termini and a C-terminal RS domain containing at least 50 amino acids with > 40% RS/SR content dipeptide repeats (Manley and Krainer, 2010; Howard and Sanford, 2015) While plant SR proteins are categorized as having one or two RRMs on the N-terminus and a downstream RS domain of at least 50 amino acids and a minimum of 20% RS/SR dipeptide repeats (Barta et al., 2010).

Certain SR proteins shuttle between the nucleus and the cytoplasm depending on their phosphorylation status. The subcellular trafficking of SR proteins is more resolved in humans, with the phosphorylation by HsSRPKs and hyperphosphorylation by CLKs being the driving force behind shuttling SR proteins from the cytoplasm to nucleus and from nuclear speckles to areas of nascent pre-mRNA (Lai et al., 2000; Ngo et al., 2005; Ghosh and Adams, 2011; Jang et al., 2019). As such their movement is highly contingent on their phosphorylation status. In plants, phosphorylation-mediated SR shuttling has also been documented (Tillemans et al., 2006; Rausin et al., 2010; Stankovic et al., 2016; Park et al., 2017). Recently, fluorescent co-localization experiments have determined that the phosphorylation of certain splicing factors by Group II AtSRPKs induced their nucleocytoplasmic shuttling (Wang et al., 2023). But the specific phosphorylation events and upstream signals/signaling pathways driving the shuttling of SR proteins to the nucleus and then to active splice sites remains to be fully characterized.

Lastly, we find that U1 snRNPs are the most highly phosphorylated snRNP group in *Arabidopsis* (**Figure 1**). U1 snRNPs are partly responsible for splice site selection (Lacadie and Rosbash, 2005; Kondo et al., 2015), inducing the ordered assembly of the remaining snRNPs to form the early and catalytic spliceosome (Cho et al., 2011). Metazoan U1 snRNP performs functions beyond pre-mRNA splicing, for instance, it is important for mRNA 3’ end cleavage (Kaida et al., 2010), polyadenylation (Ashe et al., 1997; Berg et al., 2012) and transcription (Chiu et al., 2018). The function of the plant U1 snRNP is not well characterized, with some evidence of human U1 snRNP interacting with SR proteins, suggesting a complex interaction for splice site selection (Chiu et al., 2018). It is conceivable that proteins involved in the fundamental steps of RNA splicing would require extensive phosphorylation to ensure accurate and timely initiation of AS.

### 1.3 Tissue specific phospho-regulation of splicing-related proteins

**Figure 2:**
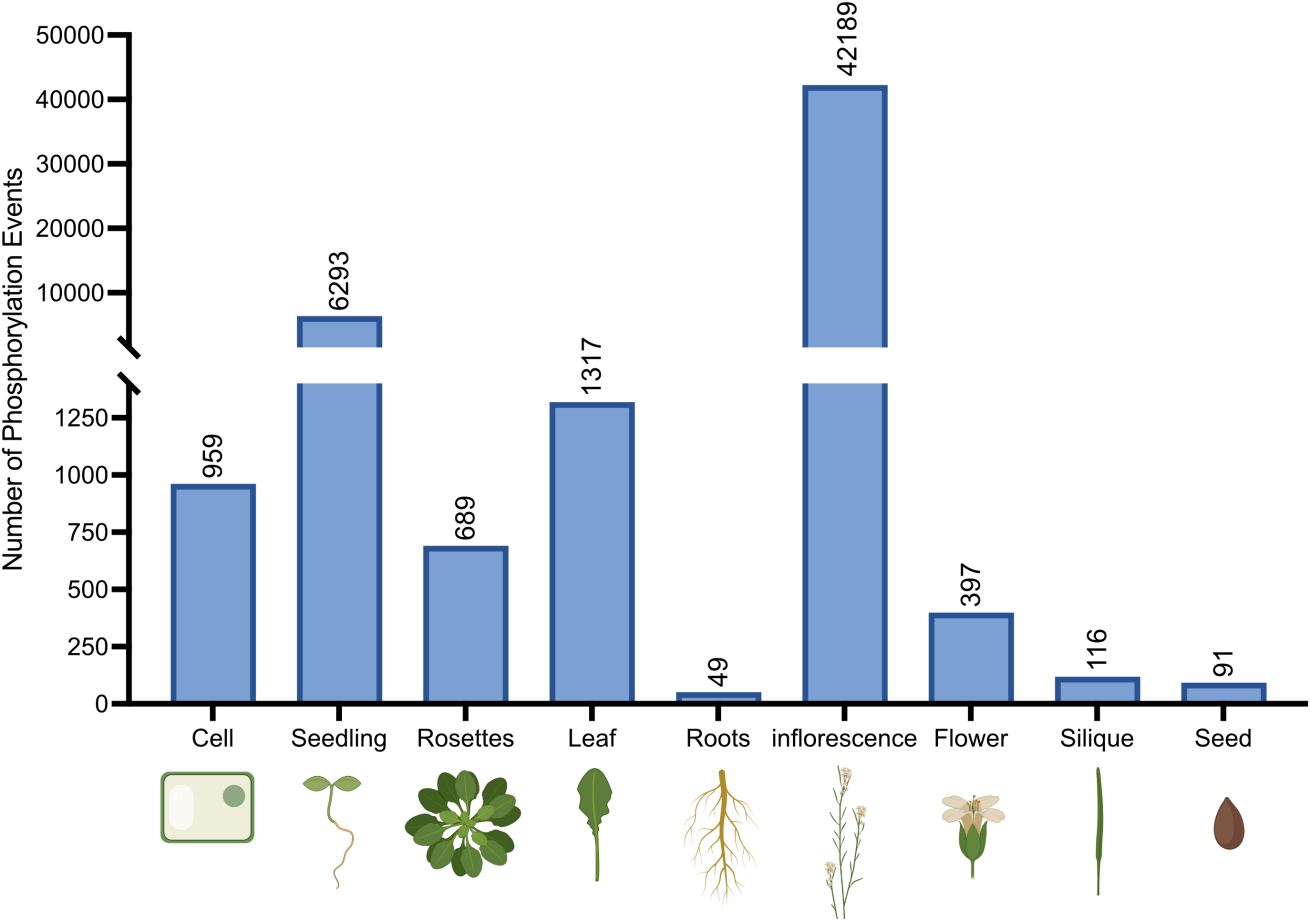
Number of unique protein phosphorylation events identified on splicing-related proteins in *Arabidopsis* tissues. Tissue-specific phosphosites were collected from PTMviewer (Willems et al., 2019; https://www.psb.ugent.be/webtools/ptm-viewer/index.php).

In humans, there is a high degree of tissue-specific AS events in which the inclusion levels of certain exons differ. Correspondingly, these AS events are termed tissue-specific (TS) exons (Clark et al., 2007; Buljan et al., 2012). Therefore, we compiled the phosphorylation events identified as occurring on splicing-related proteins based on tissue type using the PTMviewer data repository (**Figure 2**). Here, *Arabidopsis* tissues related to reproduction (inflorescences and flowers) exhibit a high degree of splicing-related protein phosphorylation. Many exogeneous and endogenous cues determine flowering timing, including: photoperiod (Kang et al., 2015; Nakamichi, 2015; Seaton et al., 2015), temperature (Lee et al., 2020a; Cao et al., 2021; Jin and Ahn, 2021), and aging (Jung et al., 2016; Hyun et al., 2017). Further, flowering is in part regulated through AS variants that either repress or promote flowering, such as FLC and CONSTANS (CO) (Park et al., 2019). The AS variants of these genes can be specifically produced in response to environmental cues and thus require finely tuned activation of specific splicing factors.

Surprisingly, root tissue was found to have the lowest number of phosphorylation events. This may be due to: 1) root tissues being under sampled in phosphoproteomic databases, or 2) regulatory differences exist in roots relative to other tissues. Interestingly however, the application of GEX1A and Pladienolide B (PB), both spliceosome specific inhibitors in humans, produced short root phenotypes in *Arabidopsis* seedlings (AlShareef et al., 2017; Ishizawa et al., 2019), suggesting spliceosome function is integral for root development. Although both studies explored the transcriptional landscape changes in inhibited tissues, neither study analyzed the phosphoproteome. Therefore, it may be possible that fewer, more integral phosphorylation events are necessary for normal root growth and development.

## 2 Concluding remarks

The study of AS and its regulation through PTMs represents an exciting new avenue of research for plant biology and plant cell regulation. Acquired proteomic data relating the intersection of protein phosphorylation and AS has gained momentum over the last five years, with the characterization of splicing-related protein kinases now emerging. Through the comparison of metazoans to plants, it is evident that many aspects of the AS regulatory machinery is *evolutionarily* conserved, however, the extent to which this machinery is *functionally* conserved remains to be uncovered.

## Author contributions

MCRG and RU contributed to the writing of this review. All authors contributed to the article and approved the submitted version.
